# A haplotype-resolved genome assembly of the Nile rat facilitates exploration of the genetic basis of diabetes

**DOI:** 10.1186/s12915-022-01427-8

**Published:** 2022-11-08

**Authors:** Huishi Toh, Chentao Yang, Giulio Formenti, Kalpana Raja, Lily Yan, Alan Tracey, William Chow, Kerstin Howe, Lucie A. Bergeron, Guojie Zhang, Bettina Haase, Jacquelyn Mountcastle, Olivier Fedrigo, John Fogg, Bogdan Kirilenko, Chetan Munegowda, Michael Hiller, Aashish Jain, Daisuke Kihara, Arang Rhie, Adam M. Phillippy, Scott A. Swanson, Peng Jiang, Dennis O. Clegg, Erich D. Jarvis, James A. Thomson, Ron Stewart, Mark J. P. Chaisson, Yury V. Bukhman

**Affiliations:** 1 Neuroscience Research Institute, University of California Santa Barbara, Santa Barbara, CA 93117 USA; 2 BGI-Shenzhen, Shenzhen, 518083 China; 3 Laboratory of Neurogenetics of Language, The Rockefeller University/HHMI, New York, NY USA; 4 Bioinformatics and Regenerative Biology, Morgridge Institute for Research, Madison, WI USA; 5 Current address: Sema4, Stamford, CT USA; 6 Department of Psychology & Neuroscience Program, Michigan State University, East Lansing, MI USA; 7 Tree of Life, Wellcome Sanger Institute, Cambridge, CB10 1SA UK; 8 Villum Centre for Biodiversity Genomics, Section for Ecology and Evolution, Department of Biology, University of Copenhagen, DK-2100 Copenhagen, Denmark; 9 State Key Laboratory of Genetic Resources and Evolution, Kunming Institute of Zoology, Chinese Academy of Sciences, Kunming, 650223 China; 10 Center for Excellence in Animal Evolution and Genetics, Chinese Academy of Sciences, Kunming, 650223 China; 11 Vertebrate Genome Lab, The Rockefeller University, New York, NY USA; 12 Department of Statistics, University of Wisconsin – Madison, Madison, WI USA; 13 LOEWE Centre for Translational Biodiversity Genomics, Senckenberganlage 25, 60325 Frankfurt, Germany; 14 Senckenberg Research Institute, Senckenberganlage 25, 60325 Frankfurt, Germany; 15 Goethe-University, Faculty of Biosciences, Max-von-Laue-Str. 9, 60438 Frankfurt, Germany; 16 Department of Computer Science, Purdue University, West Lafayette, IN USA; 17 Department of Biological Sciences, Purdue University, West Lafayette, IN USA; 18 Genome Informatics Section, National Human Genome Research Institute, Bethesda, MD USA; 19 Center for Gene Regulation in Health and Disease (GRHD), Cleveland State University, Cleveland, OH USA; 20 Department of Biological, Geological and Environmental Sciences (BGES), Cleveland State University, 2121 Euclid Ave, Cleveland, OH 44115 USA; 21 Center for RNA Science and Therapeutics, School of Medicine, Case Western Reserve University, 10900 Euclid Avenue, Cleveland, OH 44106 USA; 22 Center for Stem Cell Biology and Engineering, Neuroscience Research Institute, Mail Code 5060, University of California, Santa Barbara, CA 93016 USA; 23 The Rockefeller University, Box 54, 1230 York Avenue, New York, NY 10065 USA; 24 Department of Molecular, Cellular and Developmental Biology, University of California Santa Barbara, Santa Barbara, CA 93106 USA; 25 Department of Cell and Regenerative Biology, University of Wisconsin School of Medicine and Public Health, Madison, WI 53726 USA; 26 Regenerative Biology Laboratory, Morgridge Institute for Research, Madison, WI 53715 USA; 27 Department of Quantitative and Computational Biology, University of Southern California, Los Angeles, CA USA

**Keywords:** Arvicanthis niloticus, Genome, Diurnal, Diabetes, Long-read genome assembly, Heterozygosity, Germline mutation rate, Segmental duplications, Retrogenes, Orthology, Positive selection

## Abstract

**Background:**

The Nile rat (*Avicanthis niloticus*) is an important animal model because of its robust diurnal rhythm, a cone-rich retina, and a propensity to develop diet-induced diabetes without chemical or genetic modifications. A closer similarity to humans in these aspects, compared to the widely used *Mus musculus* and *Rattus norvegicus* models, holds the promise of better translation of research findings to the clinic.

**Results:**

We report a 2.5 Gb, chromosome-level reference genome assembly with fully resolved parental haplotypes, generated with the Vertebrate Genomes Project (VGP). The assembly is highly contiguous, with contig N50 of 11.1 Mb, scaffold N50 of 83 Mb, and 95.2% of the sequence assigned to chromosomes. We used a novel workflow to identify 3613 segmental duplications and quantify duplicated genes. Comparative analyses revealed unique genomic features of the Nile rat, including some that affect genes associated with type 2 diabetes and metabolic dysfunctions. We discuss 14 genes that are heterozygous in the Nile rat or highly diverged from the house mouse.

**Conclusions:**

Our findings reflect the exceptional level of genomic resolution present in this assembly, which will greatly expand the potential of the Nile rat as a model organism.

**Supplementary Information:**

The online version contains supplementary material available at 10.1186/s12915-022-01427-8.

## Background

**Fig. 1:**
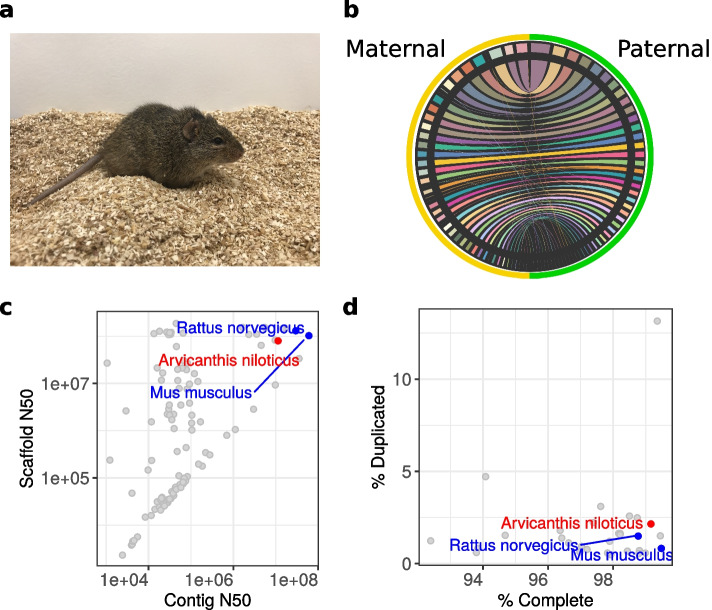
Nile rat genome assembly. **a** The Nile rat (*Arvicanthis niloticus*). **b** Scaffolded chromosomes in the maternal and paternal assemblies. Ribbons show similarities between sequences. In order to assess their heterozygosity spectrum, the assemblies have been modified from their GenBank versions as described in Materials and Methods. These modifications are documented in [3]. **c** The contig N50 values of Nile rat (*Arvicanthis niloticus*, red), house mouse (*Mus musculus*, blue), Norway rat (*Rattus norvegicus*, blue), and 106 other rodent genomes deposited in GenBank. **d** Assembly completeness evaluated using BUSCO scores, demonstrating high completeness and average percent duplicated genes that are anticipated to be single-copy genes in rodent genomes

Model organisms are essential tools for the mechanistic understanding of human physical and mental health. The high-quality genomes of house mouse (*Mus musculus*) [1] and Norway rat (*Rattus norvegicus*) [2] have enabled researchers to discover important molecular mechanisms in biological processes that have been applicable to human health. However, a wide range of human traits are not appropriately modeled by these commonly-used nocturnal rodents. The Nile rat (Fig. 1a), also known as the Nile grass rat or African grass rat, is a promising diurnal model organism to address the translational gap between animal research and human biology, particularly in two areas—circadian rhythms and type 2 diabetes.

Both house mouse and Norway rat are nocturnal, while humans are diurnal. The difference between the two chronotypes is more complex than a simple flip in daily activity pattern and likely involves distinct wiring of neural circuit and gene-regulatory networks [4–6]. Nile rats naturally exhibit clear diurnal patterns in behavior and physiology [4] and have retinal anatomy as well as large retinorecipient areas in the brain typical for animals active during the daytime [7, 8]. This makes the Nile rat an important model organism for metabolic, cardiovascular, inflammatory, neurological, and psychiatric disorders in which circadian disruption is a risk factor and/or a hallmark symptom [9].

Additionally, the Nile rat has been developed as a model of type 2 diabetes. Nile rats live without diabetes on their native diet comprising mainly grass stems and leaves [10] or laboratory high fiber diets [11, 12]. However, they rapidly develop diet-induced diabetes when fed a conventional energy-rich laboratory rodent chow [13]. Importantly, house mouse and Norway rat are relatively resistant to diet-alone induced diabetes, modeling pre-diabetes or early-diabetes and unable to develop long-term diabetic complications [14, 15]. Conversely, Nile rats on rodent chow, without chemical or genetic manipulations, recapitulate the natural progression of type 2 diabetes in humans [16] including clinically relevant diabetic complications [17–19]. Notably, the diurnal Nile rat has a cone-rich retina that is useful to study human retinal diseases, including diabetic retinopathy. The majority of laboratory housed Nile rats, including those living with diabetes, can live past 18 months whereas the reported lifespan for this species in the wild is up to 20 months for females [20].

The lack of a genome sequence has hindered the use of the Nile rat as a model organism to study molecular mechanisms of health and disease. Therefore, we initiated the Nile rat genome project within the rigorous framework of the Vertebrate Genomes Project (VGP) [21]. Here, we present a reference genome of the Nile rat, the first high-quality diurnal rodent genome with two complete haplotype-resolved parental genome assemblies. The assemblies are represented by chromosome-scale scaffolds with very few gaps. Over 30 thousand genes and pseudogenes, including sequence-resolved gene duplications, have been annotated. We used this reference genome with additional muroid genomes, in particular the house mouse, for comparative genomics analyses to identify sequences putatively associated with diet-induced diabetes. Our findings further demonstrate how haplotype-resolved assemblies and comprehensive gene annotations enable the exploration of structural and coding sequence evolution [22]. This high quality genomic assembly will greatly expand the usability of the Nile rat as a diurnal model organism.

## Results

### Nile rat assembly is highly complete, contiguous, and accurate

The principal Nile rat individual was sequenced using PacBio continuous long reads for generating contigs, and 10X Genomics linked reads, Bionano optical maps, and Hi-C proximity ligation reads for assembling contigs into scaffolds. Both parents of this individual were sequenced using Illumina short read technology and used to bin the child reads into their respective haplotypes before assembly (Table 1). The assembly, scaffolding, and quality control were performed according to VGP protocols [21]. Two sets of haplotype-resolved contigs, paternal and maternal, were generated from PacBio data using TrioCanu [23] and scaffolded using 10X Genomics, Bionano Genomics, and Hi-C data (Fig. 1b). The paternal haplotype was manually curated to reconstruct and identify chromosomes, and to correct misassemblies and remove false duplications [24]. The primary pseudohaplotype assembly used for genome annotation consisted of the paternal assembly plus the curated maternal X chromosome. Consistent with the published karyotype [25], it contained 30 autosomal super-scaffolds and 2 sex chromosomes. In total, the primary assembly contained 2.4 Gb of chromosome-level scaffolds, with an additional 1534 small unplaced scaffolds. This assembly is highly contiguous (Table 2), with a scaffold N50 of 83 Mbp and a contig N50 of 11 Mbp, one to three orders of magnitude more contiguous than murine genomes assembled using short-reads [26, 27] (Fig. 1c). The assembly is also accurate at base level, with Q value of 41 for the diploid assembly meeting the VGP standards [21].

**Table 1:** Characteristics of sequencing data. Sequencing coverage and read length of data used to assemble the Nile rat genome. The 10X genomics sequencing coverage is read coverage and not physical (read cloud) coverage. The Bionano genomics coverage counts single-molecule optical maps

Technology	Coverage	Average length
PacBio CLR	60.9	11,659
Illumina (trio)	49.3	150
HiC	68.52	N/A
10X Genomics	76.49*	N/A
Bionano Genomics	129	154,446

**Table 2:** Assembly statistics

	Principal	Maternal
Genome size	2.50 Gb	2.49 Gb
Number of scaffolds	1595	1610
Scaffold N50	82.7 Mb	81.2 Mb
Number of contigs	3219	3135
Contig N50	11.1 Mb	8.9 Mb
Percent repeat	34.4	34.32
NCBI protein-coding genes	22,234	N/A
TOGA gene annotations	21,038	21,284

The BUSCO (Benchmarking Universal Single-Copy Orthologs) annotation references the fraction of genes expected to occur in a single copy in all members of a phylogenetic group and highlights both the completeness and relative abundance of possible false duplications in an assembly [28]. The Nile rat BUSCO Complete score is 99% on the Glires subset of OrthoDB version 10 [28]. We examined the PacBio read depth over duplicated BUSCO genes to see if these duplications were correctly resolved or spurious based on sequencing coverage, setting a permissive threshold of half the average sequencing depth (30.5) to ensure high recall despite fluctuations in mapped coverage. Under this metric, 65% (285/439) are likely correctly assembled. Of the remaining annotated duplications, 63% (97/153) are assembled on unscaffolded contigs and are consistent with higher fragmentation for repetitive sequences known to be problematic for de novo assembly. Compared to other rodent genomes, the Nile rat genome assembly has superior contiguity and BUSCO completeness (Fig. 1c, d).

A total of 47.2% of the genome is composed of repetitive DNA, as determined using a combination of de novo and repeat-library based approaches [29, 30], similar to mouse (47.0%) and Norway rat (49.6%) when the same computational pipeline is applied. Repeat content identified by RepeatMasker is summarized in Table 3. The assembly was annotated with the NCBI RefSeq eukaryotic annotation pipeline [31], which identified 31,912 genes and pseudogenes, including 22,234 protein-coding genes. Additionally, we used PhyloPFP [32] to predict Gene Ontology (GO) terms for all RefSeq proteins [33].

**Table 3:** Repeat content of haplotype assemblies. Repeat masking is performed using the rodentia repeat library and RepeatMasker

Repeat	Paternal		Maternal	
	Number of elements	Percent genome	Number of elements	Percent genome
SINE	1097531	6.02	1095360	6.03
Alu/B1	449074	1.98	447915	1.98
B2-B4	584908	3.81	583853	3.82
IDs	34995	0.1	34992	0.1
MIRs	27964	0.13	28010	0.13
LINEs:	489920	12.26	488452	12.21
LINE1	474324	12.15	472927	12.11
LINE2	11535	0.08	11496	0.08
L3/CR1	2636	0.02	2598	0.02
LTR	801475	11.23	797822	11.18
ERVL	70619	0.89	70801	0.9
ERVL-MaLRs	355720	3.96	355437	3.99
ERV_classI	53595	0.8	52958	0.8
ERV_classII	318728	5.55	315822	5.48
Satellite	59080	0.28	58663	0.28
Simple repeat	1289416	2.76	1284006	2.75
Low complexity	149866	0.39	149072	0.39
Total (including other)		34.4		34.32

We used TOGA (Tool to infer Orthologs from Genome Alignments) [34] with human and mouse as references to annotate genes in the Nile rat genome. In addition to providing gene annotations, TOGA distinguishes between intact genes and genes with missing sequences or inactivating mutations, which can be used to evaluate the quality of genome assemblies. We compared the Nile rat to the genomes of 41 other species of *Muroidea* available from NCBI*.* For each of the 42 muroid genomes, we assigned 18,430 ancestral placental mammal genes to three categories: those that (1) had intact reading frames, (2) had inactivating mutations, or (3) had missing sequence parts or were completely missing from the assembly (Supplementary Table 1). Our Nile rat assembly is in third place by the number of intact ancestral genes, with 17,149 compared to 17,282 in the model organism *Mus musculus*. Additionally, an excess of inactivating mutations (e.g., frameshifting indels) in ancestral genes can be an indicator of low assembly base-level accuracy. There is no indication that Nile rat has an excess of genes with such mutations in comparison to other rodents: it ranks 12th, while some species, e.g., *Ellobius lutescens* or *Peromyscus eremicus*, have twice as many of them.

### Compilation of type 2 diabetes associated genes

This genome assembly allows us to discover sequence variations, some of which may modify gene functions. Because our group uses Nile rat to study type 2 diabetes, we developed a list of genes broadly relevant for this disease. This list was compiled from gene-disease databases [35, 36], GWAS catalog from EMBL-EBI [37], and two different text-mining methods [38, 39], resulting in a total of 4396 genes (Additional file 1: Fig. S1) [40–47]. Of these, 3295 had orthologs identified in the Nile rat assembly annotation by NCBI Orthologs database. The genes of interest were ranked according to the strength of their association with type 2 diabetes. This allowed prioritization of candidate genes in subsequent investigations of genetic variation in the Nile rat genome, including heterozygosity, gene duplication, and positive selection.

### Heterozygosity spectrum of Nile rat, an outbred laboratory species

The Nile rat colony used in this study were descendants of 29 wild Nile rats from Kenya [48], which had been bred in laboratories since 1998. Therefore, these laboratory Nile rats should have an allelic diversity largely reflective of an outbred population. Sequencing a father-mother-offspring trio provides information on genetic heterozygosity (Table 4 and Additional file 1: Fig. S2). Regions of genetic heterozygosity may suggest evolutionary flexibility to environmental adaptations. Thorough examination of heterozygosity requires both paternal and maternal haplotypes for comparison of homologous chromosomes based on whole-genome alignment. Since the Nile rat paternal and maternal haplotypes were near complete, we could detect heterozygous variants with high confidence. Next, we compared the numbers of heterozygous variants in Nile rat with those in other mammals. For the principal individual, the rate of single nucleotide variant (SNV) heterozygosity was estimated (by mapping short reads from the same individual) to be 0.086%, about 1/12 of the 1.06% rate estimated across the full spectrum of genetic variants. These estimates are similar to those from the genome assembly of another model organism, the common marmoset, created using a similar VGP trio pipeline [22]. The number of deletions and insertions are approximately equal, as expected, when comparing two parental haplotypes. We detected 626,683 small deletions (< 50 bp) and 4612 structural variant (SV) deletions (> 50 bp), in addition to 626,036 small insertions and 4632 SV insertions in Nile rat, consistent with other species [22, 23, 49]. The distribution of SV by size has several peaks in the length distribution of SVs (Additional file 1: Fig. S3), especially 300 bp, 500 bp in indels, and 300 bp, 4.5 kb in other SVs, which matches the common SV sizes of annotated transposable elements, and is consistent with the overall repeat content in the genome (Table 3).

**Table 4:** Genetic variation between the maternal and paternal assemblies

Class	Number
SNV	2,512,582
Small indel (<= 50 bp)	1,252,719
Large indel (> 50 bp)	9244
Inversion	53
Translocation	95
Inverted translocation	62
Copy number variant	1971

Comparing the two assemblies, we detected 2.51 million SNVs, with 81% of them confirmed by short-reads. More than one third of all SNVs (862,428) were located within protein-coding genes, and of those, 12,884 SNVs (10,743 SNVs validated by read mapping) were within coding exons. Two thousand nine hundred thirty-two SNVs (30%) resulted in nonsynonymous amino acid substitutions affecting 1581 genes. Iso-Seq data validated 212 of these SNVs in 208 genes, of which 42 were found in our diabetic gene list, exemplified by *Alms1* and *Slc19a2*. Human *ALMS1* and *SLC19A2* genes are both involved in monogenic diabetes disorders. Mutations in *ALMS1* can cause Alström syndrome, an autosomal recessive disorder that affects multiple organs where patients typically develop type 2 diabetes in childhood or adolescence [50]. Six heterozygous SNVs in Nile rat *Alms1* were validated by testis Iso-Seq data. One of them is *Alms1* 2256P>L, which corresponds to human *ALMS1* 3209P>L, scored as “probably damaging” by PolyPhen and “deleterious” by SIFT [51]. Out of 161 mammalian orthologs of *ALMS1*, only three have serine instead of proline in this position, implying that this residue is very well conserved. Similarly, certain *SLC19A2* mutations cause Thiamine Responsive Megaloblastic Anemia syndrome, characterized by diabetes, hearing loss, and anemia [52]. We found one SNV in Nile rat *Slc19a2*, 275R>W, confirmed by brain Iso-Seq data. This variant was not reported in UniProt, although another variant, 275R>L, was listed in ClinVar as “associated with monogenic diabetes with uncertain significance.”

### Germline mutation rate

From our trio sequencing data, we could also estimate the germline mutation rate. We found four de novo candidate mutations, with one mutation of maternal origin and three mutations of paternal origin, suggesting, as in other mammals, a male bias in the contribution to germline mutations. Accordingly, we estimated a de novo mutation rate of 0.15 × 10^−8^ mutations per site per generation, though an accurate species-level estimate would require additional samples.

### Segmental duplications

Long-read assemblies are known to resolve repetitive DNA [21, 53]. Long, low-copy repeats called segmental duplications (SD) are a class of repetitive DNA that are particularly impactful to phenotypes because they can change gene copy number and reorganize regulatory sequences [54]. We used a combination of self-alignments [55] and excess mapped read-depth to quantify SDs in the Nile rat and compared them against the long-read assemblies of the C57BL strain of house mouse [56], Norway rat, and white-footed mouse as an outgroup [57], as well as the mouse reference genome (GRCm38). When applied to the GRCm38 mouse genome assembly, our approach found abundances of SD similar to the existing annotations. Additionally, we found similar abundances of SD in GRCm38 and in the long-read assembly of the C57BL strain.

A total of 123 Mb (4.9% of the genome) of the primary assembly of the Nile rat are annotated as SD, while 114Mb (4.7%) of the maternal assembly are annotated as SD. There are 14.4 Mb of duplications assigned to Y-chromosome scaffolds, indicating there are at least 5.4 Mb of duplicated sequences that differ between parental autosomal chromosomes. Based on excess read depth, 81–106 Mb of additional sequence are missing from the combined diploid assembly due to collapsed duplications.

### Recent segmental duplication activity in Nile rat

**Fig. 2:**
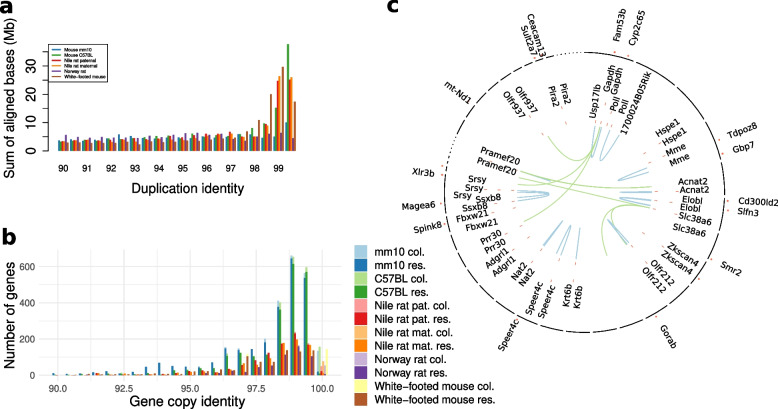
Segmental duplication content in Nile rat and related species (We used the following genome assembly versions: house mouse mm10 = GRCm38 [59], house mouse C57BL = ASM377452v2 (a PacBio long reads-based assembly [56, 60]), Norway rat mRatBN7.2 [61], and white-footed mouse UCI_PerLeu_2.1 [62]). **a** The total bases annotated as segmental duplication by SEDEF. The total includes all pairwise alignments after filtering for common repeats. **b** The total number of multi-exon genes duplicated in each of the assemblies for resolved (res.) and collapsed (col.) genes. **c** Organization of duplicated genes in the Nile rat. Tandemly duplicated genes are in blue; interspersed are in green. Genes in collapsed duplications are indicated as dots in the perimeter. The chromosomes are ordered according to genomic scaffold accessions

The genomes of all four muroid species have a high proportion of SD with high identity, indicating a recent burst of segmental duplications, consistent with previous observations in the house mouse [26, 58] (Fig. 2a). For the Nile rat, assuming three generations per year, and de novo mutation rate of 0.15 × 10^−8^, roughly 0.5% divergence accumulates every 100,000 years, suggesting that 46% of duplications (64 Mb) are younger than 200k years.

### Duplicated genes in Nile rat

We identified gene duplications based on both multi-mapping of entire gene bodies (filtering on sequence identity) and annotation of collapsed copies from excess read depth. To be conservative, multi-mapped isoforms were counted as duplications only if they contained multiple exons spanning at least 1 kb (54k distinct isoforms), with alignments of at least 90% identity and 90% of the original gene length. The high percent identity of most duplicated genes reflects a recent burst of segmental duplications in rodents (Fig. 2b). This scheme identified 403 and 369 distinct duplicated genes in the paternal and maternal assemblies respectively; of these, 84/80 were over 99.5% identical, indicating the duplication events occurred within ~100k years of the present. An additional 13/6 genes are in collapsed regions, indicating there are missing high-identity copies in the assemblies [63, 64].

**Fig. 3:**
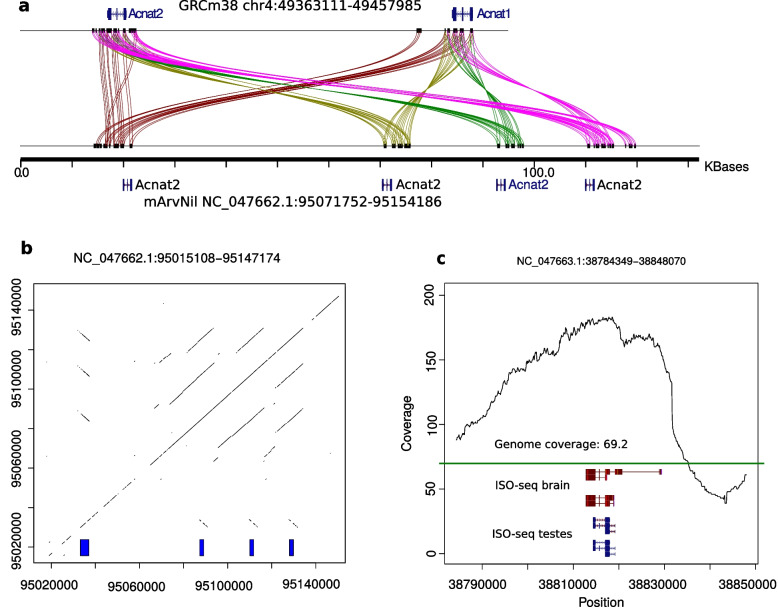
Examples of duplicated genes in Nile rat. **a** An expansion of the *Acnat2* gene in Nile rat relative to house mouse. Lines are drawn using miropeats [66], with spurious matches outside of gene bodies removed. Colors are used to emphasize gene paralogs. **b** A dot-plot of the *Acnat2* locus in Nile rat, with gene copies indicated by the blue rectangles. **c** Read-depth over *Slfn3* in the Nile rat paternal assembly. The average read depth is shown in green, indicating up to four missing copies. The gene is mapped using RefSeq GRCm38 annotations, with support from PacBio Iso-Seq reads. The gene is associated with immune response, a category of genes that often have large copy-number diversity

Of duplicated genes with known function, the most common type is olfactory (11.5–18.1%), known to exist as a dense high-copy gene cluster [65]. Additionally, 19.7–20.5% are predicted genes with unknown functions (Additional file 1: Fig. S4). Of the remaining duplicated genes in the paternal assembly, 21 are in high-identity duplications (> 99.5% identity) with at least 3 copies. Many of these are known to be of high copy number as part of large gene families or mitochondrial genes with many nuclear paralogs. These include the following: *Flg*, 10 copies; *Gapdh*, 6; *Magea*2, 7; *Magea6*, 4; *Pramef* (paralogs *6*, *17*, *20*, and *25*), 4-5; and *Ssxb* (paralogs *1-6*, *8-10*), 5. The high-quality assembly enables analysis of the mode of expansion of duplicated genes (Fig. 2c). For example, some genes were found to have been amplified in tandem arrays, e.g., *Elobl*, 5 copies; *Tdpoz9*, 3; and *Acnat2*, 4 (Fig. 3a, b), while others, such as *Slc38a6*, 5 and *Srsy*, 3, are interspersed duplications. The gene *Slfn3* is entirely mapped within a collapsed duplication, with up to three copies missing from the assembly (Fig. 3c).

*Acnat* genes arose as duplications of *Baat* genes and likely encode bile acid conjugating enzymes. These genes reside in a highly dynamic locus with multiple gene duplications and gene loss across mammalian species [67]. Whereas *Acnat* has two copies in the house mouse, our genome reveals four copies in the Nile rat (Fig. 3a). This copy number expansion in the Nile rat may affect fatty acid metabolism [68] and the synthesis of lipokines [69], which may, in turn, have implications for susceptibility to diabetes [70].

### Nile rat has fewer copies of amylase compared to the house mouse

**Fig. 4:**
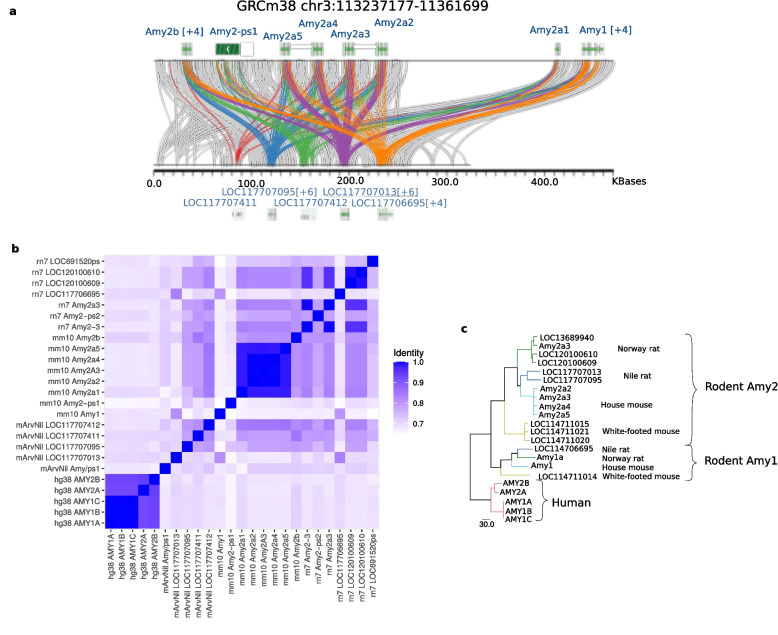
Amylase gene cluster. **a** The sequence homology in the amylase locus for mouse (top) and Nile rat rendered using miropeats. The five TOGA annotations of amylase are each rendered using separate colors. The blue and purple copies show amylase-2 homologies, orange is amylase-1, and red/green lines are annotated pseudogenes. **b** Pairwise similarity of amylase genes in human, Nile rat, mouse, and Norway rat, ordered according to their genomic coordinates. **c** A phylogenetic tree using COBALT multiple sequence alignment of amylase proteins from each of the four genomes in **b** and white footed mouse

Obesity is a comorbidity with type 2 diabetes [71] and has been found to associate with amylase-1 copy number [72]. Individuals from human populations with high-starch diets tend to have more copies of *Amy1* [73]. The amylase locus in the house mouse genome contains seven protein coding genes - *Amy1*, five copies of *Amy2a* (*Amy2a1-Aym2a5*), and *Amy2b*, as well as one amylase-like pseudogene. Our approach to annotating resolved gene duplications did not detect duplicated copies of amylase in Nile rat. However, RefSeq annotations, as well as TOGA projections of human and mouse genes to the Nile rat genome, annotate a cluster of three protein coding amylase genes, two amylase-2 and one amylase-1, plus two amylase-like pseudogenes (Fig. 4a). The two amylase-2 genes share 81.7% sequence identity across the genomic intervals, while the mouse amylase copies range from 98.6 to 99.9 percent identity with each other (Fig. 4b). Furthermore, a multiple sequence alignment of amylase proteins from Nile rat and three other rodents, house mouse, Norway rat, and white-footed mouse, shows the Nile rat amylase-1 clustering with amylase-1 proteins of the other three species, while amylase-2 copies from each species form separate clusters (Fig. 4c). This indicates that multiple amylase-2 genes are the result of recent expansions that happened independently in different muroid lineages. The two amylase-2 copies in Nile rat are more divergent from each other than any two of the four full length copies in the house mouse, perhaps reflecting the latter’s recent adaptation to commensalism with humans. Independent amylase copy number bursts have previously been reported in house mouse, Norway rat, and other species of mammals with recent adaptations to high-starch diets [74]. A relatively low amylase copy number and ancient divergence of the existing copies reflect the lack of such an adaptation in Nile rat, consistent with its natural diet, comprising mostly of grass [75].

### Extensive duplication of Ybx3-like retrogenes

**Fig. 5:**
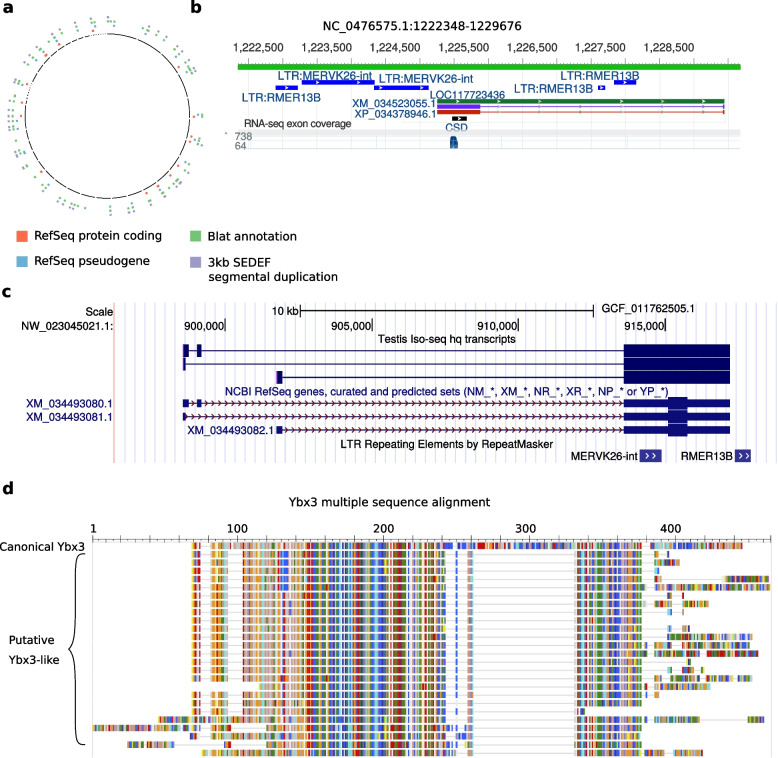
*Ybx3-like* elements in the Nile rat genome. **a** *Ybx3-like* elements are interspersed throughout the genome. Many have been annotated as protein coding genes or pseudogenes by NCBI. Most are recognized as segmental duplications by SEDEF. **b** The architecture of a typical *Ybx3-like* gene, *LOC117723436*, visualized in the NCBI Genome Data Viewer. This gene has one large and one small exon. The large exon is flanked by MERVK26-int and RMER13B endogenous retroviral elements. It contains a CSD domain and is partially supported by short read RNA-seq data. **c** Expression of *LOC117701283* in testis visualized in the UCSC genome browser. The three Iso-seq transcripts have identical CDSs, represented by thick boxes. MERV26-int is located in the 5′ UTR region, rather than outside the large exon like in most other *Ybx3-like* genes. **d** Multiple alignment of predicted *Ybx3-like* proteins and the canonical *Ybx3*, visualized by NCBI COBALT. This visualization uses the Rasmol color scheme, where amino acids with similar properties are shown in matching colors. The canonical protein is in the first row

Y-box binding proteins are a major group of cold shock proteins defined by the presence of a cold shock domain (CSD), which has DNA- and RNA-binding capabilities. Mammals have a family of three paralogous Y-box binding proteins - *Ybx1*, *Ybx2*, and *Ybx3*. Among its diverse biological roles, *Ybx3* is involved in nutrient sensing, a function commonly dysregulated in metabolic diseases. It controls the intracellular levels of large neutral and aromatic amino acids [76], including the branch chained amino acids associated with insulin resistance and obesity [77]. The NCBI genome annotation pipeline found 56 “Y-box-binding protein 3-like” (*Ybx3-like*) genes and pseudogenes in the Nile rat genome, 26 of which were annotated as protein-coding, while a BLAT search of the canonical *Ybx3* transcript against the genome found 147 hits, all dispersed throughout the genome (Fig. 5a).

*Ybx3-like* genes consist of a single large exon and often one or two small exons. The large exon is consistently flanked by two endogenous retroviral elements (ERVs), MERVK26-int upstream and RMER13B downstream, often with more than one copy of each (Fig. 5b). SEDEF annotated 78 segmental duplications that map to these genes, averaging 3.3 kb in length. These duplications encompass the large exon and the flanking ERVs. The large exon of all *Ybx3-like* genes annotated as protein-coding by RefSeq contains the CSD. One *Ybx3-like* gene, LOC117701283, is supported by three full length transcripts found in our testis Iso-seq dataset (Fig. 5c). The canonical *Ybx3* in Nile rat has 9 exons. An alignment of the predicted *Ybx3-like* proteins to the protein product of the canonical gene shows that their large exon contains most of the canonical sequence, with the exception of a large gap and the C-terminal region (Fig. 5d). The gap contains a portion of exon 5 and most of exon 6 of the canonical protein and the missing N-terminal region contains a portion of exon 8 and the entire exon 9. Many *Ybx3-like* proteins also contain N-terminal and/or C-terminal segments that are not homologous to canonical *Ybx3.*

### Duplicated Nile rat genes that exist as single copy genes in house mouse

Gene copy numbers often vary both between species and between individuals within a species. We identified 117 genes that had two or more copies in both Nile rat haplotypes while being present in a single copy in both house mouse assemblies. Of the genes duplicated in Nile rat but not in house mouse, 21 were in our diabetic gene set including *Gckr* and *Fndc4*. *Gckr* encodes glucokinase regulatory protein, which forms an inhibitory complex with glucokinase thereby regulating uptake and storage of dietary glucose [78]. Mammalian *Gckr* is composed of two sugar isomerase (SIS) domains which contain binding sites for fructose-6-phosphate (F6P) or fructose-1-phosphate (F1P) and glucokinase, where the fructose metabolites alter the affinity of *Gckr* for glucokinase [79]. Human *GCKR* has been reported as a diabetic susceptibility gene by several studies [80–84]. We found a full-length second copy of *Gckr,* LOC117716845, 1.2 Mb downstream of the canonical *Gckr*. This copy of *Gckr* has been annotated as a protein coding gene by Ensembl, ENSANLG00005017071, but RefSeq annotated it as a pseudogene. It is a high identity copy (96.7%) and both SIS domains are present. However, the F1P binding site in the first SIS domain [45] is affected by non-synonymous substitutions (Additional file 1: Fig. S5). Additionally, a BLAT search of the Nile rat canonical *Gckr* transcript yielded 25 hits. Among them, there were four other, truncated copies of high identity (92–94%) multi-exon glucokinase regulator protein-like pseudogenes. We did not detect any *Gckr* duplications in white-footed mouse or Norway rat, suggesting that this is a copy number gain in Nile rat, rather than a loss in the house mouse. A similarly duplicated gene found in Nile rat but not house mouse is *Fndc4*, where the second copy is almost full-length and validated by testis Iso-Seq data, with 97.8% identity and is located 1.3 Mb downstream of the canonical *Fndc4*. Fndc4 attenuates hyperlipidemia-induced insulin resistance in mice [85].

### Differences in protein coding gene content between Nile rat and house mouse

We used TOGA [34] to project protein coding genes from human and house mouse to the Nile rat genome. Overall, 99.7% of TOGA annotated genes in the paternal assembly are also annotated in the maternal assembly using mouse gene models; when using human gene models the number is 96.6%.

We compared TOGA projections from the mouse to genes predicted by the NCBI genome annotation pipeline and explored the genes that differ between the two species. Five hundred sixteen mouse genes appeared to be missing from Nile rat, in so far as TOGA was not able to project them to the primary haplotype assembly [86]. However, the majority of these genes were present in the assembly of the other haplotype. Examination of two examples that were part of our diabetic gene list revealed that structural variation and assembly errors were the primary reasons to not identify a gene. For example, *Hadh* is partially disrupted due to a gap in the primary haplotype assembly of the Nile rat but appears intact in the alternate (Additional file 1: Fig. S6). A different case is represented by orosomucoid 2, *Orm2.* Orosomucoid is a potential diabetes biomarker [87]. A cluster of four *Orm* genes in house mouse, including *Orm1*, *Orm2*, *Orm3*, and the pseudogene *Gm11212*, corresponds to a single gene in Nile rat, annotated by RefSeq as *Orm1*. TOGA has mapped *Orm1* and *Orm3*, but not *Orm2*, to the primary haplotype of the Nile rat. Cactus alignments confirmed the existence of a four-fold duplication in house mouse compared to Nile rat in this locus in both Nile rat haplotypes (Additional file 1: Fig. S7). Conversely, 1601 Nile rat genes annotated by NCBI could not be projected to mouse genes [88]. Most were members of families of duplicated genes, including retrogenes derived from *Ybx3* and ribosomal proteins. A gene set enrichment analysis of these genes is discussed in the Supplement (Additional file 1: Fig. S8).

There were 218 mouse genes that TOGA was unable to map to either of the Nile rat haplotypes [89], ten of which were in our diabetes gene list [90]. The two top-ranked of these, *Hmga1b* and *G6pd2*, are retrogenes that have emerged in the mouse lineage from parental genes *Hmga1* [91] and *G6pd* [92], respectively (Additional file 1: Figs. S9 and S10).

Conversely, 69 genes were absent in the house mouse and present in both Nile rat haplotypes. Seven of these genes were in our diabetic gene list [93] including *Aqp10*. Although *Aqp10* is a protein coding gene in the Nile rat and human, it is present as a nonfunctional pseudogene in the mouse [94]. Human *AQP10* has been suggested to be a target for obesity and metabolic diseases [95] but could not be studied in the house mouse where the gene has been pseudogenized.

### Positively selected genes

We identified 119 positively selected protein coding genes in Nile rat, comparing it with eight other species in the *Myomorpha* suborder via the branch-site model implemented in PAML (v4.9j), using human as an outgroup (Supplementary Table 2, [96]). To avoid confounding effects dependent on assembly quality and isoform differences, protein coding genes from all species were re-annotated using exonerate v2.4 [97]. After filtering, 7492 high quality orthologous genes remained in this dataset [98]. Out of these genes, 26 had human orthologs previously found to have low tolerance to mutation, where ≤ 20% of expected loss-of-function variants were observed in population-scale exome sequencing data annotated by gnomAD [98]. Of these 26 genes, *Xiap*, *Ppp2r5e*, *Krt1*, *Pik3r5*, and *Irf5* had amino acid substitutions in the Nile rat that did not exist in any other rodents with NCBI annotated genomes.

Here, we take a closer look at two of these genes. X-linked inhibitor of apoptosis protein (*XIAP*) prevents apoptosis of islet β-cells and is considered as a therapeutic target against β-cell destruction in diabetes [99]. *XIAP* is strongly intolerant to sequence variations, with only 2 out of 16.3 expected loss-of-function SNVs observed in humans [98]. In the Nile rat, we found three sites that were under positive selection, at positions 122, 135, and 190 within the protein sequence. Residues 135 and 190 are well-conserved across mammalian genomes. The absence of human variants in corresponding positions and the presence of nearby disease variants could indicate that mutations of these residues are consequential (Supplement). Like *XIAP*, protein phosphatase 2 regulatory subunit B’epsilon, *PPP2R5E*, is also a diabetic gene associated with pancreatic islets [100]. In humans, loss-of-function variants of *PPP2R5E* have not been reported, and only 31% of expected missense SNVs were observed [98]. In the Nile rat, we found a *Ppp2r5e* 269 I>L substitution, whereas isoleucine at this position is universally conserved across all other mammals [101].

## Discussion

The Nile rat is diurnal, has a cone-rich retina, and develops diet-induced diabetes without chemicals or genetic manipulation. Hence, the Nile rat model can complement biomedical research done in laboratory rodents lacking these characteristics. We have generated a highly contiguous, haplotype-resolved genome assembly of this species. A haplotype-resolved assembly can enable a more complete annotation by virtue of having two distinct assemblies to work with. For example, an incomplete *Hadh* gene in the paternal assembly is resolved in the maternal assembly. While the BUSCO duplication value appears to be higher than many other rodent assemblies, a read-depth analysis suggests that 65% of redundant BUSCOs are likely actual duplicated genes, indicating that the BUSCO gene annotations may be improved as additional high-quality genomes are analyzed.

A haplotype-resolved assembly enabled us to explore all types of heterozygosity, including SNVs and SVs (indels, and other structural polymorphisms of all sizes). Overall, we observed a level of heterozygosity consistent with an outbred organism. However, chromosomes 1, 3, and 5 had large regions of low heterozygosity. These may have resulted from inbreeding of close relatives that occurred at generation 4 due to small colony size, although no direct brother-sister matings were used. The sequenced individual is from generation 6.

Because a trio pedigree was sequenced for the Nile rat, we were also able to calculate the rate of de novo germline mutations. This rate is 0.15 × 10^−8^ mutations per site per generation, lower than the mutation rates reported for other mammals [102]. A more accurate, population-based estimate can be a subject of future research.

This assembly enabled us to resolve most segmental duplications and catalog multicopy genes. However, some collapses remain, which encompass 6–16% of all multicopy genes. While advances in technology and assembly algorithms will reduce the number of collapsed duplications, annotations of genes in collapses should be continued until an approach guaranteeing telomere-to-telomere assembly is established.

A comparative analysis of the Nile rat with other rodents enabled us to detect several types of evolutionary events affecting genes associated with type 2 diabetes (Table 5). We selected ten genes for a closer examination. *Gckr* [78], *Fndc4* [103], *amylase*, and *Orm* are differently affected by segmental duplications in Nile rat and house mouse. *Hmga1b* and *G6pd2* have been created by retrotransposition in the mouse. Multiple copies of *Ybx3-like* genes have been created in Nile rat by retrotransposition followed by segmental duplication, similar to *TP53* in elephants [104]. *Alms1* and *Slc19a2* are affected by heterozygosity, and *Xiap* by positive selection in the Nile rat lineage.

**Table 5:** Copy number divergent, heterozygous, and positively selected genes

Category	Number of genes	Linked to type 2 diabetes	Comment
Human-house mouse orthologs	18,951	3567	Based on the MGI orthologs table
Human-Nile rat orthologs	16,235	3295	Based on the NCBI Gene database
Duplicated in mouse but not in Nile rat	368	15	
Duplicated in Nile rat but not in mouse	117	21	
Mouse genes missing in Nile rat	218	10	Homozygous-missing only
Named Nile rat genes missing in mouse^a^	69	7	See footnote
Non-synonymous SNVs supported by Iso-seq	208	42	
Positively selected	119	19	

^a^Assigned names other than locus numbers by NCBI, no mouse genes mapped by TOGA, and names do not occur in the MGI table of human-mouse orthologs

## Conclusions

We have presented a reference-quality, haplotype-resolved genome assembly of the Nile rat Arvicanthis niloticus. We have performed several types of analysis to characterize the Nile rat genome, compare it to related species, and identify potential drivers of susceptibility to diet induced diabetes.

Retrotransposition and segmental duplication are major drivers of genome evolution, including creation of new genes. Comparing reference-quality assemblies of closely related species enabled us to observe these events at high levels of detail. Evaluating the ability of these new genes to express functional proteins and their impacts on the biology of the Nile rat necessitates future studies. We hope that the availability of a reference-quality genome of this important species will both inspire and enable future research.

## Methods

### Nile rat tissue collection

Spleen, brain, and testis tissue were collected from a 21-week-old male Nile rat (T564M) in the laboratory colony of Huishi Toh and James Thomson at University of California, Santa Barbara. The spleen was used for genome sequencing, whereas the brain and testis were used for transcriptome analysis. These tissue samples were flash frozen in liquid nitrogen immediately after dissection. Additionally, blood samples were collected via cardiac puncture from Nile rat T564M’s parents (T480F and T469M). T564M is a generation 6 descendent of 17 breeders that were imported from the Hayes lab in Brandeis University, a secondary colony of the original laboratory colony from the Smale lab in Michigan State University, which started from 29 wild Nile rats captured in Kenya. The Nile rats in this study were fed a high fiber diet 5326 and were normoglycemic.

### Genome sequencing

#### Primary subject

We isolated 23 μg of ultra-high molecular weight DNA (uHMW) from 35 mg of flash-frozen spleen tissue using the agarose plug Bionano Genomics protocol for animal tissue (DNA isolation fibrous tissue protocol #30071C). uHMW DNA quality was assessed by a Pulsed Field Gel assay and quantified with a Qubit 2 Fluorometer. Ten micrograms of uHMW DNA was sheared using a 26 G blunt end needle (Pacbio protocol PN 101-181-000 Version 05). A large-insert Pacbio library (CLR) was prepared using the Pacific Biosciences Express Template Prep Kit v1.0 (#101-357-000) following the manufacturer protocol. The library was then size selected (> 20 kb) using the Sage Science BluePippin Size-Selection System. The Pacbio library was sequenced on 22 PacBio 1M v3 (#101-531-000) SMRT Cells on a Pacbio Sequel instrument using the sequencing kit 3.0 (#101-597-800) and a 10-h movie. A total of 206.97 Gb of raw reads data with an average insert size N50 of 23,715 bp bases was generated. Unfragmented uHMW DNA was used to generate a linked-reads library on the 10X Genomics Chromium (Genome Library Kit & Gel Bead Kit v2 PN-120258, Genome Chip Kit v2 PN-120257, i7 Multiplex Kit PN-120262). From this 10X library, we generated 256.78 Gb of sequence data on an Illumina Novaseq S4 150bp PE lane. uHMW DNA was labeled for Bionano Genomics optical mapping using the Bionano Prep Direct Label and Stain (DLS) Protocol (30206E) and run on one Saphyr instrument chip flowcell. Hi-C preparation was performed by Arima Genomics using the Arima-HiC kit (P/N: A510008), and an Illumina-compatible library was generated using the KAPA Hyper Prep kit (P/N: KK8504). This library was then sequenced on an Illumina HiSeq X (150bp PE) at 129X coverage following the manufacturer’s protocols. Sequencing read lengths and depths of coverage are summarized in Table 1.

#### Parents

PCR-free Illumina libraries were generated from 1 μg genomic DNA using a Covaris LE220-plus to shear the DNA and the TruSeq® DNA PCR-Free HT Sample Preparation Kit (Illumina) for library generation. The median insert sizes were approximately 400 bp. Libraries were tagged with unique dual index DNA barcodes to allow pooling of libraries and minimize the impact of barcode hopping. Libraries were pooled for sequencing on the NovaSeq 6000 (Illumina) to obtain at least 750 million 151-base read pairs per library. This resulted in 49.3X coverage of the parental genomes.

### Transcriptome sequencing

We extracted and purified total RNA from brain and testis tissues using the QIAGEN RNAeasy kit (Cat. No. 74104). For each tissue, 25–30 mg was cut into 2mm pieces before homogenization with the Qiagen TissueRuptor II (Cat No./ID: 9002755). The quality of all RNAs were assessed using a Fragment Analyzer (Agilent Technologies, Santa Clara, CA) and quantified with a Qubit 2 Fluorometer (Qubit™ RNA BR Assay Kit - Catalog number: Q10210).

PacBio Iso-seq libraries were prepared according to the ‘Procedure & Checklist - Iso-Seq Template Preparation for Sequel Systems’ (PN 101-070-200 version 05). Specifically, cDNA was reverse transcribed using the SMRTer PCR cDNA synthesis kit (Clontech, Mountain View, CA) from 329 ng and 374 ng of total RNA for brain and testis respectively. Amplified cDNA was cleaned with AMPure beads and a PacBio library was prepared using the Pacific Biosciences Express Template Prep Kit v1.0 (#101-357-000) following the manufacturer protocol. PacBio Iso-seq libraries were sequenced on a PacBio Sequel (sequencing chemistry 3.0) with 20 h of movie time. We sequenced one SMRT Cell for each Iso-seq library. We then used the Iso-seq application in the PacBio SMRT Link package to generate Circular Consensus Sequences (CCSs), remove cDNA primers and concatemers, identified strandedness, trim polyA tails, and perform de novo clustering and consensus call to output high-quality full-length consensus isoforms.

### Genome assembly and annotation

The haplotype-resolved assembly was generated using TrioCanu v. 1.8 using the parental Illumina reads and the PacBio WGS data (Koren et al. 2018). Consensus sequences were generated using Arrow v. smrtlink_6.0.0.47841 (Pacific Biosciences), followed by purging of spurious duplications using purge_dups v. 1.0.0 [105]. The assemblies were then scaffolded using 10X Genomics linked long reads with scaff10x v. 4.1.0, Bionano optical maps with Solve v. 3.2.1_04122018, and HiC data with Salsa2 HiC v. 2.2. The scaffolds were polished using PacBio reads with Arrow and 10X Genomics synthetic long reads with Longranger and Freebayes v. 1.3.1. This was followed by decontamination and manual curation [24]. The mitochondrial genome was assembled using mitoVGP workflow v2.0 [106].

The genome was annotated using the RefSeq eukaryotic annotation pipeline [107] with 73,241 brain and testes Iso-Seq full-length transcript sequences [108]. There were 457,991 isoforms in 21,723 distinct coding regions. The quality of the consensus was sufficiently high that the majority of annotated gene models were complete; 2.7% of genes (591/21,723) required modification of the reference to account for frameshift errors.

We used Phylo-PFP [32] to assign Gene Ontology (GO) terms to protein coding genes. Phylo-PFP is a sequence-based protein function prediction method which mines functional information from a broad range of similar sequences, including those with a low sequence similarity identified by a PSI-BLAST search. The sequences retrieved from PSI-BLAST are reranked by considering the phylogenetic distance and the sequence similarity to the query. Incorporating phylogenetic information leads to better functional similarity estimation. Gene Ontology (GO) terms of each retrieved protein are assigned the same score as the sequence. Finally, for each GO term, scores from all sequences are summed. The prediction is also enriched with GO terms that have greater than 90% probability of co-occurrence.

### Assembly quality metrics

In order to evaluate the quality of our assembly, we compared it to representative genomes of other species of rodents available from the NCBI assembly database. We utilized R package rentrez, a wrapper for NCBI E-utilities, to retrieve assembly records. The R script used for the retrieval and plotting of assembly quality metrics is available on OSF [109].

The *Q* value of the diploid assembly was computed using the merqury software [110], with k-mer databases built using 10X reads for the child and Illumina reads for the parents [111].

### Segmental duplication analysis

The annotation pipeline is available from [112]

#### Segmental duplication annotation with self-alignments

Genomes were repeat masked using the union of windowmasker v1.0.0 and RepeatMasker 4.1.1 with the parameter “-species rodentia.” An initial set of segmental duplications were identified using SEDEF version 1.1-37-gd14abac-dirty with default parameters (Numanagic et al. 2018) that were then filtered in post-processing to remove mobile elements annotated as segmental duplications. First, duplications were removed if either copy was over 90% repeat masked. Next, the remaining annotations contained duplications that were 1–2 kb, high copy (> 20 copies) and were typically masked as endogenous retroviruses using the CENSOR repeat masking server [113]. To remove these, high-copy duplications were detected and filtered from the duplication set. The multiplicity of a duplication was measured considering transitive copies potentially missed in alignments by creating a graph where every repeated interval corresponds to a node, and edges connect both the pair of nodes corresponding to the repeat alignments, and any overlapping intervals. The number of unique intervals in each connected component was used to assign a repeat copy number, and repeats with copy number greater than 20 were removed.

#### Gene duplication annotation

Duplicated genes were annotated using multi-mapped sequences. Gene models were defined using Nile rat RefSeq sequences aligned using minimap2 using the -x splice option. Next, sequences of genes with at least one intron with a gene body of at least 1 kb were mapped back to each assembly using minimap2. Alignments with at most 10% divergence that were at least 90% of the query sequence length were considered as duplicated genes. A single isoform for each gene was retained as a duplication. When multiple genes map to the same location, only the first sequence mapped by the pipeline is retained. The number of copies of a gene are counted in the resulting set of alignments.

#### Annotation of collapsed repeats

We used a hidden Markov model to assign copy numbers to collapsed duplications. Each copy number is encoded as a hidden state from 0 to a maximum of 12 copies. The observed data are the coverage values in 100-base bins across each assembly. The probability of emission is calculated as a negative binomial with a mean and variance estimated according to the copy number of each state based off of the mean observed at the copy-number two sites in the genome.

### Mutation rate analysis

The offspring and parental reads were mapped to each assembly independently (paternal and maternal). Duplicate reads and reads mapping to more than one region were removed. Variants were called using GATK 4.0.7 HaplotypeCaller in base-pair resolution mode, calling each single site of the genome. Two independent joint genotyping analyses were carried out: one for the three individuals (mother, father, offspring) mapped to the maternal assembly and one for the three individuals mapped to the paternal assembly. The variant file was filtered on the quality of the genotyping features following these parameters: QD < 2.0, FS > 20.0, MQ < 40.0, MQRankSum < -2.0, MQRankSum > 4.0, ReadPosRankSum < -3.0, ReadPosRankSum > 3.0, SOR > 3.0.

Additional filters were applied at each position to detect the candidate mutations. Thus, a site would be filtered out if one individual had:

- A depth DP < 0.5 × depth _individual_ and DP > 2 × depth _individual_, with depth _individual_ being the average depth of the individual (depth _individual_ offspring: 56 X, depth _individual_ father: 78 X and depth _individual_ mother: 84 X)
- A genotype quality GQ < 60
- A number of alternative alleles in the parents with AD > 0
- An allelic balance in the offspring with AB < 0.3 and AB > 0.7

We then identified the maternal de novo candidates using the following genotypes:

- Sites where the parents are homozygous for the reference (0/0) and the offspring is heterozygous (0/1) when mapped to the paternal genome: 35 candidates
- Sites where the parents are homozygous for the alternative (1/1) and the offspring is heterozygous (0/1) when mapped to the maternal genome: 133 candidates

A comparison of the reads in the candidates’ sites resulted in only one position with an overlap of read names. Thus, we found one maternal de novo candidate mutation.

Similarly, we identified the paternal de novo candidates using the following genotypes:

- Sites where the parents are homozygous for the reference (0/0) and the offspring is heterozygous (0/1) when mapped to the maternal genome: 38 candidates
- Sites where the parents are homozygous for the alternative (1/1) and the offspring is heterozygous (0/1) when mapped to the paternal genome: 143 candidates

The comparison of reads in both datasets resulted in three positions with overlapping reads. Thus, we retained three paternal de novo candidate mutations.

To estimate a per generation rate, we calculated callability, the number of sites with full detection power. These were all the sites that passed the DP, the GQ, and the AD filters. The maternal callability was 1,371,536,436 base pairs, and the paternal callability was 1,365,805,112 base pairs. This callability estimation does not take into account the filters applied only on polymorphic sites that could have reduced the detection power on some of the callable sites. To correct for any bias due to the site filters and the allelic balance filter we applied a false negative rate (FNR) correction on the callability. The FNR was calculated as the number of true heterozygous sites, i.e., one parent homozygous for the reference allele, one parent homozygous for the alternative allele and the offspring heterozygous, filtered out by the AB filter. This FNR also took into account the proportion of callable sites expected to be filtered out by the site filters if a variant was present. FNR was ~5% on both the maternal and paternal assembly.

Finally, we estimated the mutation rate using a diploid genome size of 2.6 Gb.

### Heterozygosity spectrum

To call heterozygous sites between the two haploid sequences, we directly compared two haploid assemblies using Mummer (v3.23) with the parameters of “nucmer -maxmatch -l 100 -c 500.” Before retrieving all spectrum of genetic variants, we refined haplotype genomes by anchoring the scaffolds which might be lost in final assemblies (Additional file 1: Fig. S11). SNV and small indels were generated by “delta-filter -m -i 90 -l 100” and followed by “dnadiff.” Several custom scripts were used to deal with Mummer output [114]. We employed Assemblytics v1.2 [115] and SyRi v1.0 [116] to detect SVs from Mummer alignment using default parameters. Specifically, Assemblytics for large indels and CNV and SyRi for inversions, translocations, and other SVs. SVs in which more than half the feature consisted of gaps were dropped.

### Branch-site test analysis

To find positively selected genes (PSGs) in the Nile rat lineage, we compared Nile Rat to eight other species of *Myomorpha*—lesser Egyptian jerboa *Jaculus jaculus*, Eurasian water vole *Arvicola amphibius*, golden hamster *Mesocricetus auratus*, white-footed mouse *Peromyscus leucopus*, Mongolian gerbil *Meriones unguiculatus*, house mouse *Mus musculus*, brown/Norway rat *Rattus norvegicus*, and human as an outgroup. To mitigate the effects of assembly quality and isoforms from different versions of assemblies, we re-annotated protein-coding genes of the 9 *Myomorpha* species by exonerate v2.4 [97] using 20,426 human gene models that were generated by selecting the longest isoform and removing the pseudogenes.

After excluding genes lost in any taxa, a total of 19,628 orthologous genes remained for protein alignment. For detecting PSGs, we tested only candidates that passed a series of rigorous filters: (1) each gene had to map to the human gene with at least 70% coverage, (2) frameshift indels in coding sequences (CDS) were prohibited, and (3) genes with premature stop codons were ruled out. A total of 7492 high-quality orthologous genes remained.

The positive selection sites in Nile rat were detected by the branch-site model using PAML (v4.9j). Genes with an FDR-adjusted p-value less than 0.05 were treated as candidates for positive selection. To minimize effects of assembly and sequence alignment, we filtered positive selective sites by the following criteria: (1) the positive selective site was a gap in more than two species and (2) the PSG sites have more than two nonsynonymous substitution forms (ignoring the outgroup). We also performed a manual check for all individual PSGs to remove any other false-positives caused by low-quality alignments. This procedure detected 119 PSGs.

### Identification of diabetes-linked genes by text mining

We used four techniques to derive a set of genes associated with type 2 diabetes and with diet-induced diabetes. First, we compiled an expert-curated gene-disease association database from standard resources, the Comparative Toxicogenomics Database [35] and PharmGKB [36]. The result gave 277 genes associated with type 2 diabetes, but none associated with diet-induced diabetes. Next, we employed Kinderminer, a simple text mining system developed to query ~32 million PubMed abstracts to retrieve significantly associated target terms (e.g., genes) for an input key phrase (e.g., type 2 diabetes, diet-induced diabetes). KinderMiner retrieved 460 genes for type 2 diabetes and four genes for diet-induced diabetes. Third, we applied Serial KinderMiner (SKiM), a literature-based discovery system (LBD) that extends KinderMiner, querying the PubMed abstracts to find C terms (e.g., genes) for an input A term (e.g., type 2 diabetes, diet-induced diabetes) via some intermediate B terms (i.e., a list of phenotypes and symptoms). The set of B terms comprised only the top 50 phenotypes and symptoms significantly associated with type 2 diabetes or diet-induced diabetes. SKiM yielded 1941 genes for type 2 diabetes and 2254 genes for diet-induced diabetes. Restriction of the SKiM run to the top 50 phenotypes and symptoms ranked based on a prediction score is commonly practiced in other existing LBD systems such as LION LBD [117] and BITOLA [118]. In SKiM, the prediction score is calculated as a sum of negative logarithmic value of Fisher exact test (FET) *p*-value and sort ratio (i.e., the number of PubMed abstracts with A and B terms divided by the number of PubMed abstracts with B terms). Finally, we used a GWAS database [37], which reported type 2 diabetes-associated SNPs in 1482 genes.

We ranked the strength of association of each gene with diabetes as follows. An association reported in the gene-disease databases received a score of 3, an association reported by KinderMiner or the GWAS database received a score of 2, and SKiM the score of 1. If a gene was reported by more than one method, the scores were added up, so that the composite score ranged from 1 to 8.

## Supplementary Information

**Additional file 1: Figure S1.** Venn diagram of gene lists linked to type 2 diabetes by different types of evidence. **Figure S2.** Heterozygosity inferred by comparing the paternal and maternal scaffolded contigs, shown on the paternal scaffolds. **Figure S3.** Length distributions of structural variants. **Figure S4.** Functional classification of duplicated genes. **Figure S5.** Sequence alignment of Nile rat Gckr proteins to 113 mammalian orthologs. **Figure S6.** Missing Nile rat Hadh gene present in alternate haplotype assembly. **Figure S7.** Orm genes duplicated in house mouse but not in Nile rat. **Figure S8.** Top20 GO terms overrepresented in Nile rat genes that do not overlap TOGA projections from house mouse. **Figure S9.** Hmga1b mouse gene absent in the Nile rat genome. **Figure S10.** G6pd2 mouse gene absent in the Nile rat genome. **Figure S11.** Schematic diagram of trimming alignment.

**Additional file 2: Supplementary Table 1.** TOGA status of 18430 ancestral placental mammal genes in muroid rodent genome assemblies. **Supplementary Table 2.** Positively selected genes and their mutation tolerance in humans according to gnomAD.

## Data Availability

*Primary genomic sequencing data* VGP GenomeArk: https://vgp.github.io/genomeark/Arvicanthis_niloticus/ *Transcriptomic sequencing data* We have deposited brain and testis Iso-seq data to the SRA: • Brain: SRX8145073 • Testis: SRX8145073 *Genome assemblies and annotation* All genome assemblies and annotations generated and used in this study are available from NCBI. Genome: https://www.ncbi.nlm.nih.gov/genome/?term=Arvicanthis+niloticus. Annotated reference genome assembly, genome browser, and other information are linked to from this page. Reference, annotated genome assembly: mArvNil1.pat.X. This assembly contains paternal haplotype with maternal X chromosome. BioProject PRJNA632612; RefSeq Assembly GCF_011762505.1. Note: This version of the assembly does not contain the mitochondrion: NCBI was unable to include it for technical reasons. The maternal assembly is RefSeq Assembly GCA_011750645.1. Nile rat genome annotation on NCBI FTP server: https://ftp.ncbi.nlm.nih.gov/genomes/all/annotation_releases/61156/100/ BioSample: SAMN12611849 Other genome assemblies in GenBank: 1. Principal pseudohaplotype: mArvNil1.pat.X. Contains paternal haplotype with maternal X chromosome. This assembly is identical to the RefSeq assembly but also contains the mitochondrial chromosome as a contig. BioProject PRJNA608735; Assembly GCA_011762505.1; mitochondrion CM022273 2. Paternal haplotype (uncurated): mArvNil1.pat. BioProject PRJNA561935; Assembly GCA_011762545.1 3. Maternal haplotype (uncurated): mArvNil1.mat. BioProject PRJNA561936; Assembly GCA_011750645.1. Note: this haplotype has not been curated. Therefore, some chromosomes remain split into 2 or more scaffolds *Genomes used in comparative analysis of segmental duplications are available from the NCBI Assembly database:* 1. mm10 house mouse assembly, https://identifiers.org/insdc.gca:GCA_000001635.8 2. C57BL house mouse assembly, ASM377452v2, https://identifiers.org/insdc.gca:GCA_003774525.2 3. Norway rat mRatBN7.2, https://identifiers.org/insdc.gca:GCA_015227675.2 4. White-footed mouse, UCI_PerLeu_2.1, https://identifiers.org/insdc.gca:GCA_004664715.2 *TOGA projections of human and mouse genome annotations to Nile rat* TOGA annotations are available at https://genome.senckenberg.de/download/TOGA/ UCSC genome browsers with TOGA annotations of the Nile rat genome are also available: • Primary pseudohaplotype (paternal + X): https://genome.senckenberg.de//cgi-bin/hgTracks?db=HLarvNil1 (mArvNil1.pat.X assembly) • Alternate haplotype: https://genome.senckenberg.de//cgi-bin/hgTracks?db=HLarvNil1B (mArvNil1.mat assembly) *Supplementary datasets* The datasets supporting the conclusions of this article are available in the OSF repository, DOI 10.17605/OSF.IO/J97KC, https://osf.io/j97kc/.

## Abbreviations

VGP: Vertebrate Genomes Project
BUSCO: Benchmarking Universal Single-Copy Orthologs
GO: Gene Ontology
SNV: Single nucleotide variant
SV: Structural variant
CDS: Coding sequence
SD: Segmental duplication
SEDEF: Segmental Duplication Evaluation Framework
PAML: Phylogenetic Analysis by Maximum Likelihood
TOGA: Tool to infer Orthologs from Genome Alignment
COBALT: Constraint-Based Alignment Tool
CSD: Cold shock domain
SKiM: Serial KinderMiner
LBD: Literature-based discovery system
FNR: False negative rate
FET: Fisher exact test
PSG: Positively selected genes
ERV: Endogenous retroviral elements
GWAS: Genome-wide association studies
NCBI: National Center for Biotechnology Information
EMBL-EBI: European Molecular Biology Laboratory-European Bioinformatics Institute
SIS: Sugar isomerase
